# The Role of the Mediterranean Diet in Breast Cancer Survivorship: A Systematic Review and Meta-Analysis of Observational Studies and Randomised Controlled Trials

**DOI:** 10.3390/nu15092099

**Published:** 2023-04-27

**Authors:** Ge Chen, Sam Leary, Jizhao Niu, Rachel Perry, Angeliki Papadaki

**Affiliations:** 1 Bristol Dental School, University of Bristol, Bristol BS2 8AE, UK; ge.chen.2019@bristol.ac.uk (G.C.); s.d.leary@bristol.ac.uk (S.L.); 2 National Institute for Health Research Bristol Biomedical Research Centre, University Hospitals Bristol NHS Foundation Trust and University of Bristol, Bristol BS8 2BN, UK; 3 Centre for Exercise, Nutrition and Health Sciences, School for Policy Studies, University of Bristol, Bristol BS8 1TZ, UK; jizhao.niu@bristol.ac.uk; 4 Bristol Medical School (Cardiovascular Surgery and Vascular Biology), University of Bristol, Bristol BS2 8HW, UK; rachel.perry@bristol.ac.uk

**Keywords:** breast cancer, Mediterranean diet, survivorship, mortality, quality of life, systematic review

## Abstract

Female breast cancer is the most frequently diagnosed cancer. The long-term survival rates for this disease have increased; however, the unique demand for high-quality healthcare to improve breast-cancer survivorship are commonly unmet. The Mediterranean diet (MD) is associated with reduced breast-cancer risk and various health-related benefits in the general population, but its effect on breast-cancer survivors remains uncertain. The objective of this systematic review and meta-analysis was to assess current evidence from randomised controlled trials (RCTs) and observational studies (cohort, cross-sectional and case-control) regarding the effect of the MD on survival, quality of life (QoL) and health-related outcomes in female breast-cancer survivors. MEDLINE, EMBASE, Web of Science and the Cochrane library were searched for studies published before and including April 2022. Two reviewers independently screened the literature and completed the data extraction and risk-of-bias assessment. Eleven studies (fifteen reports) were included, including two RCTs, four cohort and five cross-sectional studies. The meta-analysis of the cohort studies showed strong evidence of an inverse association between high adherence to the MD and all-cause mortality (hazard ratio (HR) 0.78, 95% confidence interval (CI) 0.66–0.93, I^2^: 0%, Grading of Recommendations Assessment, Development and Evaluation (GRADE) = low certainty of evidence) and non-breast-cancer mortality (HR 0.67, 95% CI 0.50–0.90, I^2^: 0%, GRADE = very low certainty of evidence). The associations between high adherence to the MD and QoL and health-related parameters were not consistent. These findings highlight the potential of adherence to the MD to reduce the risk of mortality. Future research with better study designs, as well as more consistent measurements of QoL and MD adherence, taking into account changes in MD adherence over time and population subgroups, is needed to provide more robust evidence on the survival, QoL and health-related outcomes in BC survivors.

## 1. Introduction

Female breast cancer (BC) is the most frequently diagnosed cancer and the leading cause of cancer mortality among women [1]. Through earlier screening and more effective treatments, long-term survival rates in BC patients have increased in recent decades, with 5-year survival rates at around 80% [2,3,4]. However, long-term effects of treatments, such as weight gain/obesity and menopausal symptoms, as well as increased risk of cardiovascular disease (CVD) and osteoporosis, are commonly experienced by BC survivors during or after completing active cancer treatments [5]. These effects may reduce compliance with prolonged cancer treatment, increase the risk of cancer recurrence, and negatively affect quality of life (QoL) for BC survivors [5]. Therefore, it is imperative to identify intervention strategies to not only improve survival rates in BC patients, but also enhance their overall QoL and prevent short- and long-term complications [6,7].

Currently, BC survivors are instructed to follow the cancer-prevention-lifestyle guidelines for the general population [8,9,10]. This is an important gap in evidence-based practice, as lifestyle advice could affect cancer survival differently to incidence risk [11]. The World Cancer Research Fund/American Institute for Cancer Research (WCRF/AICR) synthesised the evidence for associations between diet, nutrition and physical activity and cancer survival from intervention and observational studies. They found that most of these studies did not account for cancer subtypes and treatments, so the specific evidence for the direct effects of lifestyle factors on BC survival is currently insufficient [11]. Moreover, most of the studies reported on specific foods or nutrients, but foods are rarely consumed in isolation. In contrast, focusing on dietary patterns could more accurately represent overall dietary quality, provide useful insights into the associations between diet and health outcomes, and present an opportunity for direct translation into clinical and public health guidelines [12,13].

The Mediterranean diet (MD) is a plant-based dietary pattern, characterised by the high intake of olive oil and plant foods (such as fruits, vegetables, non-refined cereals, legumes and nuts), low-to-moderate intake of dairy products, fish and poultry, moderate intake of alcohol, and low intake of red meat and sweets [14]. This dietary pattern could reduce BC risk and improve BC survival via anti-inflammatory effects, antioxidant properties and hormone–receptor interactions [15,16,17]. A recent meta-analysis of 23 observational studies found that high adherence to the MD was inversely associated with the risk of BC (risk ratio (RR) 0.94, 95% confidence interval (CI) 0.90–0.97) in the general population, as well as with the risk of all-cause mortality (RR 0.75, 95% CI 0.66–0.86) in cancer survivors [18]. However, only 110 BC cases out of 4883 cancer cases were included in the pooled analysis of all-cause mortality [18,19], which was insufficient to quantify the effect of MD on BC survivors.

Moreover, studies have reported the beneficial effects of following a MD on various health-related outcomes in the general population, such as reduced risk of cardiovascular disease [20], self-reported menopausal symptoms [21] and hip fracture [22]. Although these outcomes are potential long-term/late side-effects of treatments for BC [5], studies conducted on BC survivors are limited and vary in their intervention components, and no study to date has systematically assessed these complications of BC treatment. To our knowledge, no systematic review has presented a comprehensive evaluation of the effect of the MD on BC survivorship. Therefore, the aim of this systematic review was to assess the evidence regarding the effect of the MD on survival, QoL and health-related outcomes in BC survivors, in order to inform further intervention development and healthcare decision-making.

## 2. Methods

The protocol of this review was registered with the International Prospective Register of Systematic Reviews (PROSPERO) database (registration no. CRD42022318559). Reporting followed the Preferred Reporting Items for Systematic reviews and Meta-Analyses (PRISMA) guidelines (Supplementary Materials Table S1) [23].

### 2.1. Selection Criteria

#### 2.1.1. Study Design

Randomised controlled trials and observational (cohort, cross-sectional and case–control) studies investigating the effect of the MD on BC-survivorship outcomes were included. Reviews, case studies and abstracts with no full-text reports available were excluded.

#### 2.1.2. Participants

Studies involving adult females (≥18 years old) who were diagnosed with BC (regardless of cancer stages, subtypes and comorbidities), including those living with BC, undergoing BC treatment and living beyond cancer [11,24] were included. Studies involving participants with other breast tumours (e.g., breast sarcoma), pregnant or lactating participants and wider population groups for which information on BC survival could not be extracted were excluded.

#### 2.1.3. Intervention/Exposure

The intervention/exposure of interest was adherence to the MD. To be included, trials needed to have promoted the MD or a Mediterranean-style diet (with or without physical activity, as long as physical activity was also equally promoted to the control group). Observational studies should have assessed adherence to the MD among BC survivors. Furthermore, due to variation in interventions of MD and measurements of MD adherence across studies, studies were included if they utilised a MD scale, such as the Mediterranean Diet Score (MDS, ranging from 0–9) [25,26], to measure adherence to the MD or a Mediterranean-style diet.

#### 2.1.4. Comparison

The comparators were defined according to the intervention/exposure. The control group in the included trials should have received either no treatment, usual care, or advice to follow a different diet (with or without physical activity, as long as physical activity was equally promoted to the intervention group). Trials were excluded if they did not have a control group, or if both the intervention and the control groups received treatments containing the MD. Observational studies were included if they reported the results of non-adherence or low adherence (compared to highest level of adherence) to the MD or Mediterranean-style diet among BC survivors, measured by the same MD scale as that used for the group with high MD adherence, to allow comparison.

#### 2.1.5. Outcomes

The outcomes of this review were identified from the Breast Cancer Survivorship Care Guidelines, produced by the American Cancer Society to assist clinical practice [5], and the common themes of cancer survivorship [7]. The primary outcomes were categorised into mortality, BC recurrence and QoL, which are key factors for addressing BC survival, health and wellness in BC survivors [7,24]. The secondary outcomes included the categories of health-related parameters, long-term/late effects of BC treatments and the safety of the intervention/exposure, which further focused on the common issues of BC follow-up care and comorbidity management [5]. The detailed outcomes are presented in Table 1.

### 2.2. Search Strategy

The literature-search strategy was developed by the lead author (G.C.) and checked by an experienced librarian. Relevant published papers were searched in the following electronic databases: (1) Embase via Ovid (from 1974 to present); (2) MEDLINE (from 1964 to present); (3) Web of Science (from 1900 to present): Science Citation Index Expanded (SCI-EXPANDED); Conference Proceedings Citation Index–Science, and; (4) Cochrane library (from inception to present).

The search strategy for the aforementioned databases was similar, but revised accordingly by using controlled vocabulary and syntax rules. Search terms were identified for each of the following domains: population (“breast cancer*” or “breast neoplasm*” or “breast tumor* or tumour*” or “breast carcinoma*”, “mammary cancer*” or “mammary neoplasm*” or “mammary tumor* or tumour*” or “mammary carcinoma*”); and intervention/exposure (“Mediterranean diet” or “Mediterranean lifestyle”). The literature search applied keywords, MeSH (Medical Subject Headings), Emtree and synonyms (and their combinations). No language restrictions were applied and non-English articles were translated where possible. The detailed search strategy is presented in Supplementary Materials Table S2.

The reference lists of systematic reviews, review articles from the primary search results and eligible papers were hand-searched to identify additional eligible original studies. The corresponding authors of conference papers and abstracts were contacted to establish whether full-text articles were available. The search was carried out in April 2022.

### 2.3. Study Records

The search results were managed using the Rayyan (http://rayyan.qcri.org, accessed on 30 March 2022) software app [27]. After eliminating duplicates, the retrieved papers were screened by two reviewers (G.C. and J.N.), independently. The titles and abstracts of the identified papers were evaluated first, and then the full texts were assessed against the inclusion and exclusion criteria. Following full-text screening, an Excel data-extraction form was designed and piloted on a sample of studies by the reviewers, and then the data of included studies were extracted by two reviewers, independently (G.C. and J.N.). Any differences of opinion between the two reviewers regarding study eligibility and inconsistencies in data were discussed with a third reviewer (R.P.) in order to reach consensus. The corresponding authors of the included studies were contacted when clarifications or other formats of data were required.

### 2.4. Quality Assessment

Risk-of-bias assessment was conducted on all primary and secondary outcomes that were included in the meta-analyses. Full texts were assessed for risk of bias by two reviewers (G.C. and J.N.), independently, and discrepancies were resolved through discussion with a third reviewer (R.P. or A.P. or S.L.) until consensus was reached. The cohort subscale of the Newcastle–Ottawa scale (maximum of 9 stars) was used to assess the risk of bias in cohort studies. Due to limited availability of risk-of-bias assessment tools for cross-sectional studies, an adapted Newcastle–Ottawa scale (maximum of 10 stars) was applied (Supplementary Materials Table S3). Both scales assessed risk of bias from three perspectives: selection of the study groups, comparability of the study groups, and determination of either the exposure or outcome of interest [28]. Risk of bias for RCTs was assessed using the Risk of Bias Version 2 tool (RoB 2) [29].

### 2.5. Data Analysis

Meta-analyses were conducted in Stata 17.0 [30] if comparable data were available from two or more studies which reported the outcomes using the same format. Results were pooled according to the adjustment for different sets of confounders, which were classified into minimal adjustment, medium adjustment and maximum adjustment, where possible. The potential confounder domains included demographic and clinical characteristics (e.g., age, education, ethnicity, smoking status, physical activity, BMI), female-specific factors (e.g., menopausal status), diet (e.g., total energy intake) and cancer (e.g., subtype, stage, treatments). Fixed-effects models were used when heterogeneity was not substantial (I^2^ statistic < 50%) [31,32]; otherwise, random-effects models were used. Effect estimates for adherence to the MD were summarised and/or pooled with their corresponding 95% CIs by using RR or hazard ratios (HR) for dichotomous outcomes and mean differences for continuous outcomes, depending on the data availability in the study report. The strength of the evidence for meta-analysed outcomes was assessed using the Grading of Recommendations Assessment, Development and Evaluation (GRADE) system (software: https://gradepro.org/, accessed on 1 October 2022) [33]. Funnel plots and Egger’s regression tests [34] (for testing publication bias), subgroup analyses (e.g., according to menopausal status and BC subtype) and meta-regression were not conducted due to low number of studies included in meta-analyses.

For results that were not suitable for statistical pooling, harvest plots were used to visually display the findings of all included studies in a matrix of bar graphs [35]. In each bar graph that comprised the matrix, the heights of the bars indicated designs of included studies (high: RCT, medium: cohort study, low: cross-sectional study), and the colours of the bars indicated whether the result of the study was included in the meta-analysis (grey: included in meta-analyses, black: not included in meta-analyses). The rows of the matrix indicated the detected associations (positive, none or negative) between MD adherence and outcomes reported by each study. The columns of the matrix indicated the outcomes reported by the included studies.

### 2.6. Sensitivity Analyses

The robustness of conclusions was examined by repeating the meta-analyses using fixed- or random-effects models, as appropriate [32], as well as using only studies classified as low-risk-of-bias if sufficient data were available.

## 3. Results

**Figure 1:**
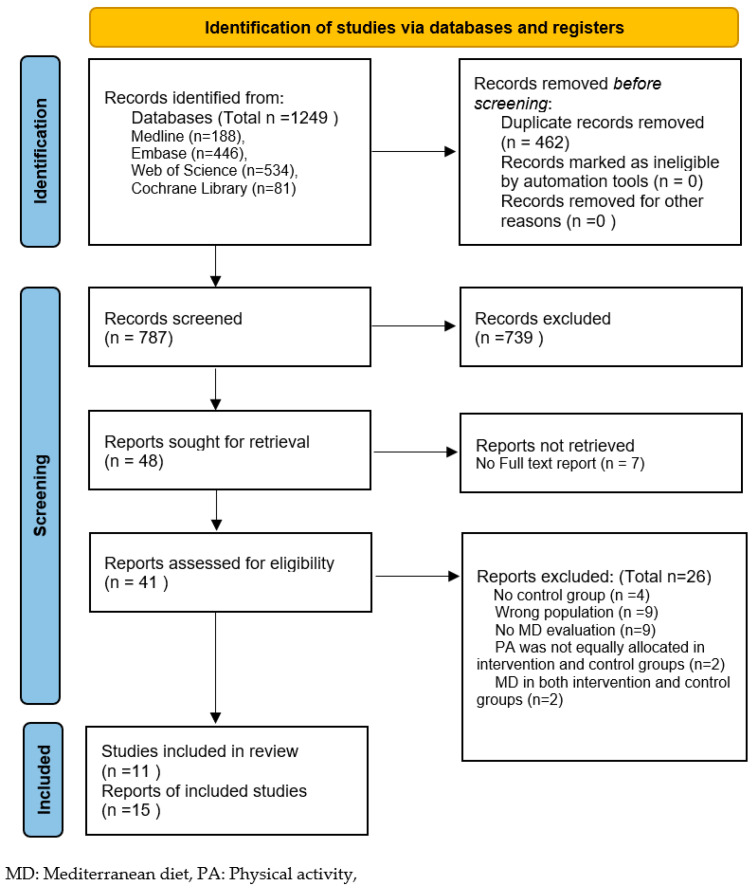
Preferred reporting items for systematic reviews and meta-analyses (PRISMA) flow diagram.

A total of 11 studies (from 15 reports) were included from the 1249 reports originally retrieved from the datasets. These consisted of two RCTs (from four reports), four cohort studies and five cross-sectional studies (from seven reports). No further studies were identified from the reference lists of review papers (*n* = 41) and included reports (*n* = 15). The selection process is presented in the PRISMA flow diagram below (Figure 1). The reasons for excluding reports at the full-text-screening stage are reported in the Supplementary Materials Table S4.

### 3.1. General Characteristics

The characteristics of the included studies are presented in Supplementary Materials Table S5. The studies were conducted in North America (*n* = 4), Italy (*n* = 4), Spain (*n* = 2) and Greece (*n* = 1). The participants were aged between 23 and 94 years, were mainly at stage 0–III (three studies also included participants at stage IV) and had finished primary cancer treatments (such as surgery, chemotherapy and radiotherapy); endocrine therapy and/or target therapy may have been used during the study period. The median percentage of participants at stages I–II was 84.0% (range 58.4–89.1%), based on seven studies, while the median percentage of participants who had estrogen- and/or progesterone-receptor-positive BC was 62.9% (range 20.0–83.9%), based on six studies.

### 3.2. Quality Assessment and Strength of Evidence

All the included studies were assessed for risk of bias for all the outcomes that were included in the meta-analyses. This was the case for all of this review’s primary outcomes (mortality, BC recurrence, QoL) and/or body-mass index (BMI). The results are presented in Table 2.

Most of the cohort studies that reported mortality (3/4) and BC recurrence (1/1) were rated as having low risk of bias. These studies included all the participants with follow-up data in the analysis and collected outcomes from medical linkage, with an average follow-up period of more than nine years. All of the cohort studies reported mortality adjusted for age in the minimally adjusted model. The maximally adjusted models varied across the studies and included variables related to demographic and clinical characteristics (education, ethnicity, marital status, smoking status, physical activity, BMI), female-specific factors (menopausal status, menopausal-hormone-therapy use), diet (total energy intake), and/or cancer (subtype, stage, period since cancer diagnosis, treatments).

The studies that reported QoL and/or BMI were predominantly cross-sectional. Most of these studies (4/5) were rated as having high risk of bias, mainly due to unjustified sample sizes, lack of information regarding non-responders and low comparability (meaning that no adjustment for confounders was applied). One RCT reported QoL and the other RCT reported BMI; both were rated as having high risk of bias, since blinding could not be used for the MD interventions and interventions were not fully implemented as stated in the study, or information was not provided regarding the intervention quality.

The details on the strength of evidence assessed by GRADE for these four outcomes are presented in Supplementary Materials Table S6.

### 3.3. Main Results

**Figure 2:**
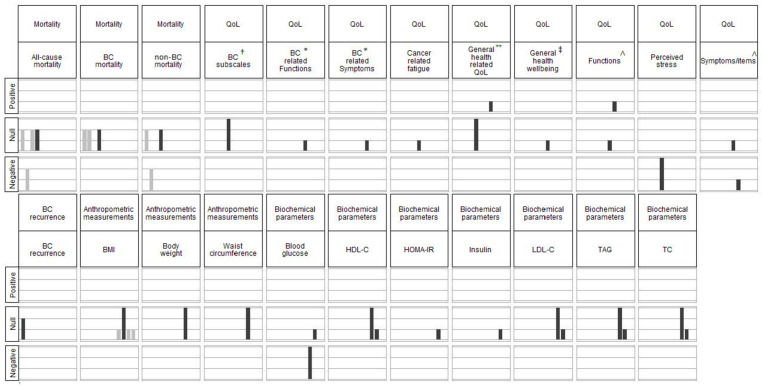
Summary of the evidence (harvest plot) from all the included studies (*n* = 11) for the associations between MD and reported outcomes. BC: breast cancer; BMI: body-mass index; HDL-C: high-density-lipoprotein cholesterol; HOMA-IR: Homeostatic Model Assessment of Insulin Resistance; QoL: quality of life; TAG: triacylglycerol; TC: total cholesterol; LDL-C: low-density-lipoprotein cholesterol. In each bar graph in the matrix, the heights of the bars indicate designs of included studies (high: RCT, medium: cohort study, low: cross-sectional study) and the colours of the bars indicate whether the results of the study were included in the meta-analysis (grey: included in meta-analyses, black: not included in meta-analyses). The rows of the matrix indicate the detected associations (positive, none or negative) between MD adherence and the outcomes reported by each study. The columns of the matrix indicate the outcomes reported by the included studies. † Breast-cancer subscales of FACT-B scale. * Subscales of EORTC QLQ-BR23 scale. ** The overall score for FACT-G or EQ-5D-3L. ‡ The Global Health Status/QoL subscale of EORTC QLQ-C30 scale. ∧ Subscales of EORTC QLQ-C30 scale.

The study characteristics and results are summarised in Table 3. The studies measured the exposure by the original or adapted 9-component MD score (*n* = 5) [25,26], 14-item PREDIMED (PREvención con DIeta MEDiterránea) scale (*n* = 5) [50] or a 0–55 diet score (*n* = 1) [51]. However, the cut-off values used to indicate high/low adherence to the MD varied between the studies. The reported associations are summarised and presented in the harvest plot (Figure 2). Overall, the reported results included mortality (*n* = 4), BC recurrence (*n* = 1), QoL (*n* = 4) and anthropometric and biochemical parameters (*n* = 4). None of the studies reported the progression of newly diagnosed cancer, the incidence of long-term/late effects or adverse events. Funnel plots, Egger’s regression tests and subgroup analyses could not be conducted due to the insufficient number of studies.

#### 3.3.1. Primary Outcomes

##### Mortality

**Figure 3:**
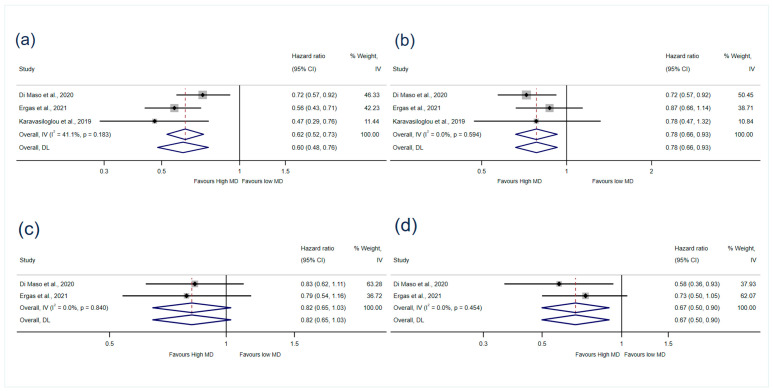
Meta-analyses of MD adherence and (**a**) all-cause mortality (minimally adjusted), (**b**) all-cause mortality (maximally adjusted), (**c**) BC mortality (medium adjusted), (**d**) non-BC mortality (medium adjusted). IV: Weights are from fixed-effects model. DL: Weights are from random-effects model. MD: Mediterranean diet [19,36,37].

Four of the cohort studies [19,36,37,38] reported associations between MD adherence and mortality (Figure 2). Three studies [19,36,37] (5223 participants) reported associations with all-cause mortality that could be meta-analysed. There was strong evidence that high MD adherence reduced the risk of all-cause mortality by 40% (HR 0.60, 95% CI 0.48–0.76, I^2^: 41.1%) after minimal adjustment and by 22% (HR 0.78, 95% CI 0.66–0.93, I^2^: 0%) after maximal adjustment for confounders (GRADE = low certainty of evidence) (Figure 3). The results when medium adjustment for confounders was applied are presented in Supplementary Materials Figure S1.

Two studies [36,37] (5113 participants) reporting the associations with BC mortality and non-BC mortality were included in the meta-analysis. The pooled results showed that high MD adherence reduced the risk of non-BC mortality (HR 0.67, 95% CI 0.50–0.90, I^2^ = 0%, GRADE = very low certainty of evidence), but there was no association with BC mortality (HR 0.82, 95% CI 0.65–1.03, I^2^ = 0%, GRADE = very low certainty of evidence) (Figure 3).

One of the cohort studies was not included in the meta-analysis as it reported RRs. It showed no association between adherence to the MD and all-cause mortality, BC mortality and non-BC mortality when applying the maximally adjusted model (Table 3) [38].

##### BC Recurrence

Only one study reported BC recurrence and no evidence was found of an association (HR 1.08, 95% CI 0.79–1.47) [36].

##### QoL

Four studies reported the association between MD adherence and QoL. These included one RCT [46] and three cross-sectional studies [39,41,42]. However, the results could not be meta-analysed due to the variation in the QoL measurements, study designs and reporting formats. As shown in Figure 2, positive associations were found between MD adherence and physical functioning and general health-related QoL (EQ-5D-3L scale) [42], whereas negative associations were found for cancer-related symptoms (pain, dyspnoea, insomnia) [42] and perceived stress [46]. No associations were found with cancer-related fatigue, BC-related QoL subscales and other domains of the FACT-G and EORTC QLQ-C30 scales [39,41,42,46].

#### 3.3.2. Secondary Outcomes

##### Anthropometric Measurements

**Figure 4:**
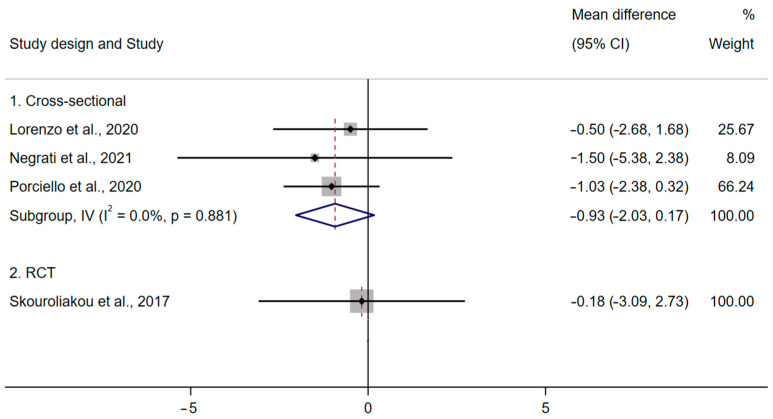
Meta-analysis of MD adherence and BMI (kg/m^2^) (fixed-effects model) [42,44,45,49].

Four studies [42,44,45,49] reported anthropometric measurements, including BMI, body weight and waist circumference (Figure 2). The results from three cross-sectional studies [42,44,45] (GRADE = very low certainty of evidence) and an RCT [49] showed no evidence that the MD has a role in BMI in BC survivors (Figure 4). There was no association between MD and body weight or waist circumference [45,49] (Figure 2).

##### Biochemical Parameters

One of the RCTs [49] and a cross-sectional study [44] reported biochemical parameters. The results indicated that the MD led to lower blood-glucose levels at 6 months, compared with the control group [49], but there were no other associations or between-group differences with regards to high-density-lipoprotein cholesterol (HDL-C), low-density-lipoprotein cholesterol (LDL-C), total cholesterol, triacylglycerol, insulin or the homeostatic model assessment of insulin resistance (HOMA-IR) (Figure 2).

### 3.4. Sensitivity Analysis

All the meta-analyses were repeated using random-effects models rather than fixed-effects models and their results were similar (Figure 3 and Supplementary Materials Figure S2). The studies that reported mortality (all-cause mortality, BC mortality and non-BC mortality) and BMI included in the meta-analyses were all rated as having a low risk of bias and a high risk of bias, respectively. Therefore, a sensitivity analysis using only low-risk-of-bias studies was not conducted.

## 4. Discussion

To our knowledge, this is the first systematic review investigating the role of the MD in BC survivorship. The study synthesised the current evidence on survival, quality of life and health-related outcomes to address key issues in BC survivorship. The multivariate pooled analysis showed strong evidence of reduced risk of all-cause mortality, with low heterogeneity (HR 0.78, 95% CI 0.66–0.93, I^2^ = 0%, GRADE = low certainty of evidence). This finding is consistent with the most up-to-date systematic review on adherence to the MD and cancer, which reported the association of the MD with all-cause mortality (RR cohort 0.75, 95% CI 0.66–0.86, I^2^ = 41%) based on cancer patients in general, but with a very limited number of BC patients (110 out of 4883 participants) [18].

Our systematic review did not find an association between MD adherence and BC mortality. Interestingly, Morze et al. [18] meta-analysed 18 cohort studies on members of the general population before cancer diagnosis and found a reduced risk of overall cancer mortality with high MD adherence (RR cohort 0.87, 95% CI 0.82–0.92; I^2^ = 83%). The substantial heterogeneity in their analysis might have been due to the varied cancer types examined, but might also imply that the pre-cancer assessment of MD adherence affects cancer mortality differently, compared with post-cancer assessment. The WCRF report on the association between diet (assessed following cancer diagnosis) and BC prognosis suggested limited evidence of an association between healthy eating patterns and reduced risk of death [58]. In this report, the MD was examined by three cohort studies [19,36,38] and measured post-diagnosis [58]. The studies noted that the reason for assessing MD adherence post-cancer-diagnosis was due to the changes in eating behaviour that might occur after cancer treatment [38] and the possibility that the use of post-diagnosis diet to improve prognosis may be of particular interest to cancer survivors [59]. Future studies could consider how diet changes over time might affect the role of MD in BC survivorship from the post-diagnosis stage.

We mostly found no associations between adherence to the MD and QoL and health-reported parameters, apart from the improved QoL and reduced blood-glucose reported by single studies. These findings might have been due to the small sample sizes of the studies examining these outcomes, meaning that these studies had less statistical power with which to detect genuine associations (*n* < 100 with no reported sample size calculations) [41,44] and/or to the studies’ application of only one cut-off point for the MD scale utilised (e.g., >7 for high adherence on the PREDIMED scale) to categorise high and low MD adherence [39,60]. The latter, in particular, might not fully reflect differences in adherence levels. In contrast, all the studies included in the all-cause-mortality meta-analysis used/adapted the nine-component MD score [26], with two relatively large studies (including 5113 participants) applying distinct cut-off points to define high (6–9) and low (0–2/3) MD adherence [36,37]. An earlier RCT on 3088 early-stage-BC survivors tested the effect of a diet high in vegetables, fruit and fibre and low in fat intake for 6 years and found no evidence to support a protective effect on BC-free survival and overall mortality at 7.3 years of follow-up [61]. This was different from our findings, which might imply that the effect of the MD is exerted through the overall dietary pattern. In order to more accurately investigate the effect of the MD rather than single foods/nutrients, we only included studies using a MD scale that quantified adherence to the MD. Higher scores measured by a MD scale could reflect better consistency with the entire MD pattern rather than several of its elements. Therefore, to establish the effect of the MD, an interval between the cut-off points of the applied MD scale for defining high and low adherence should be considered in future research.

Previous research noted that cancer survivorship includes acute, extended and permanent phases, including the period of cancer diagnosis/primary treatment, dealing with the consequences of treatment after its completion and living with cancer as a chronic disease, respectively [24,62]. Thus, we included studies in which participants had been diagnosed with BC, regardless of the cancer stage and subtype. Our intention was to conduct subgroup analyses to investigate the differences between the associations according to factors such as menopausal status and BC subtype, since these factors significantly affect BC treatment plans and survival, as well as BC patients experiencing different long-term/late effects and QoL as a result of the treatments they received. These subgroup analyses could not be conducted due to the insufficient reporting of results in the included studies. Our findings were based on the majority of participants, who were at an early stage (I–II), overweight or obese (the BMI was, on average, above 25 kg/m^2^), with limited information in terms of cancer subtype and menopausal status. Thus, our findings cannot be applied to all BC survivors and should be interpreted cautiously, taking into account the reviewed studies’ participants’ characteristics.

The GRADE assessment [33] of our findings only showed low-to-very-low certainty of evidence. This was mainly due to the potential unmeasured and residual confounding of data from the observational studies that were included in the meta-analyses. Nevertheless, three cohort studies included in the mortality meta-analysis and one cross-sectional study reported improved QoL results [19,36,37,42] after adjusting for the main confounders (age, total energy intake, education, cancer stage, subtypes, menopausal status). These studies were assessed as having medium-to-low risk of bias, which provided results of relatively good quality. Although well-designed, sufficiently powered RCTs can be assessed as presenting higher strength of evidence, large long-term RCTs that evaluate nutrition exposure may not always be feasible to conduct to test causality [63,64]. The RCTs included in our systematic review only implemented short-term (up to 12 months) interventions, which might not be sufficient to reflect the true effect of adherence to the MD. These issues could be addressed in future studies by applying causal inference methodologies, such as Mendelian randomization (MR), which uses genetic variations that are strongly associated with putative environmental risk factors (e.g., dietary factors) to evaluate the possible causal relationships between these factors and disease outcomes [65,66].

The strengths of this review include the fact that it systematically synthesised the evidence on the role of the MD in BC survivorship for the first time. This review applied rigorous performance and reporting methods by following the Cochrane Handbook and the PRISMA guidelines [23,67]. Further, we used the ROB 2/Newcastle–Ottawa scale and the GRADE system to assess the risk of bias of the included studies and the strength of the evidence, respectively. In addition, we examined the MD as a whole, plant-based, dietary pattern, instead of individual foods or nutrients. This is important because exploring the role of whole dietary patterns enables the possibility of direct translation to clinical practice [12,13]. Furthermore, this study examined the effect of the MD on both the short- and the long-term outcomes of BC survivorship based on current evidence, by including experimental studies and observational studies. More importantly, we explored the outcomes of QoL and other health-related parameters to address aspects of cancer survivorship, in addition to survival, which provided a more comprehensive understanding about the role of the MD in BC survivorship.

This study also had several limitations. First, this review could not establish the effect of the MD on the long-term or late effects of cancer treatment, nor of its effect on different subgroups of BC survivors, such as those with BC subtypes and those with different menopausal statuses, due to the lack of studies reporting this information with detailed results. Further, not all the findings from the studies included in this review could be meta-analysed (e.g., QoL). This was mainly because the studies applied different designs and scales (e.g., FACT-G and EORTC-QLQ-30), so the pooling of their results could have been misleading [68,69]. However, the narrative summary and harvest plot provided important insights on these associations. In addition, the time of MD assessment and the MD-adherence cut-off points varied across the studies. Considering the possibility of participants making dietary changes over time, the current findings may not fully reflect the true exposures, as the assessment of the MD was only based on a single measurement at baseline and different cut-off points were used to define high/low MD adherence. Future studies should consider categorising MD adherence by changes over time and/or applying clear cut-off points to further understand its effect on BC survivorship. Moreover, causality cannot be inferred from the current findings, since the synthesised evidence was derived mostly from observational studies and graded from low to very low certainty by GRADE. Finally, the included studies were predominantly conducted on populations in Europe and the US. Therefore, our results may not be generalisable to other populations.

## 5. Conclusions

In conclusion, this systematic review found that high adherence to the MD is associated with reduced risk of all-cause mortality and non-BC mortality. Although these findings were rated as having low-to-very-low certainty by GRADE, they highlight the potential long-term benefits of adhering to the MD, at least for the outcomes related to BC survivorship in this study. No consistent associations were found between high adherence to the MD and QoL and health-related parameters. Future research with better study designs, more consistent measurements of QoL and MD adherence and the consideration of changes in MD adherence over time and population subgroups, is needed to provide more robust evidence on the survival, QoL and health-related outcomes of BC survivors.

## Figures and Tables

**Table 1:** Outcomes of the current systematic review.

	Categories of Outcomes	Outcomes Included
Primary outcomes	Mortality	All-cause mortality; BC mortality; non-BC mortality.
	BC recurrence and/or newly diagnosed cancer progression	e.g., local recurrence; metastasis.
	Quality of life	Measured by generic or cancer-specific validated scales, (e.g., Functional Assessment of Cancer Therapy—Breast Cancer).
Secondary outcomes	Health-related parameters	Biochemical parameters, (e.g., blood glucose and blood lipids, including total cholesterol, low-density lipoproteins, high-density lipoproteins, triglycerides).
		Anthropometric measurements, (e.g., body-mass index, waist circumference, body weight).
	Incidence of long-term/late-effect BC treatments	Osteoporosis/osteopenia/fracture; obesity/cardiovascular disease/stroke/diabetes; endometrial cancer/other secondary cancer (primary cancer); cognitive impairment; mortality from cancer treatments.
	Adverse events	

BC: breast cancer.

**Table 2:** Risk-of-bias assessment of included studies.

Observational Studies		Assessment Tool: Newcastle–Ottawa Scale (Cohort Studies)					
	Outcome	Selection (Maximum 4 *)	Comparability (Maximum 2 *)	Outcome (Maximum 3 *)		Overall Stars	Overall Risk
Ergas et al., 2021 [36]	BC Recurrence	****	**	***		9/9	Low
	Mortality	****	**	***		9/9	Low
Di Maso et al., 2020 [37]	Mortality	****	*	***		8/9	Low
Karavasiloglou et al., 2019 [19]	Mortality	****	*	***		8/9	Low
Kim et al., 2011 [38]	Mortality	**	*	*		4/9	High
**Assessment Tool: Newcastle–Ottawa Scale** **(Adapted for Cross-Sectional Studies)**							
		**Selection** (Maximum 5 *)	Comparability (Maximum 2 *)	**Outcome** (Maximum 3 *)	**Overall Stars**		
Alvarez-Bustos et al., 2021 [39]; (Ruiz-Casado et al., 2020 [40])	QoL	**		**		4/10	High
Barchitta et al., 2020 [41]	QoL	**		*		3/10	High
Porciello et al., 2020 [42]; (Porciello et al., 2019 [43])	QoL	***	*	**		6/10	Medium
	BMI	***		**		5/10	High
Negrati et al., 2021 [44]	BMI	**		***		5/10	High
Lorenzo et al., 2020 [45]	BMI	**		***		5/10	High
**RCTs**		**Assessment Tool: Risk of Bias Version 2**					
	**Outcome**	**Randomisation Process**	**Deviations from Intended** **Interventions**	**Missing** **Outcome Data**	**Measurement** **of the** **Outcome**	**Selection of the Reported Result**	**Overall Risk**
Long Parma et al., 2022 [46]; (Zuniga et al., 2019 [47]; Ramirez et al., 2017 [48])	QoL	Low	High	low	Some concerns	High	High
Skouroliakou et al., 2017 [49]	BMI	High	High	High	Some concerns	Some concerns	High

BMI: body-mass index; QoL: quality of life; RCT: randomised controlled trial; *, **, ***, ****: star.

**Table 3:** Study characteristics and results by reported outcomes (order by outcomes, study design and author).

Mortality and BC Recurrence									
Study	Country of Study	Study Design	Sample Size/Number in Analysis	Dietary Assessment and/or MD Adherence Assessment	Duration/ Follow-Up	Exposure	Comparator	Main Result (HR/RR, 95% CI)	Variables Used for Adjustment
Di Maso et al., 2020 [37]	Italy	Cohort	1453/1453 (<5% missing data on BMI and education)	FFQ (2 years before BC diagnosis) and 9-component MD score [26]	Truncated 15 years after diagnosis (cohort follow-up median: 12.6 years; maximum: 16.8 years)	MDS: 6−9	MDS: 0−3	All-cause mortality: HR-adjusted: 0.72 (0.32–0.92) Breast-cancer mortality: HR-adjusted: 0.83 (0.62–1.11) Non-breast cancer mortality: HR-adjusted: 0.58 (0.36–0.93)	Age (at diagnosis), total energy intake, years of education, menopausal status, TNM stage, ER/PR status, area of residence and calendar period at diagnosis, (BC and non-BC mortality further adjusted for competing risk according to Fine–Gray model)
Ergas et al., 2021 [36]	USA	Cohort	4505/3660	FFQ and aMED-diet score (adapted from 9-component MD score) [26]	Recruit 2005–2013, end of follow-up in December 2018, mean: 9.08 years (SD 2.77)	aMDS: 6–9	aMEDS: 0–2	Breast-cancer recurrence: HR model 2: 1.08 (0.79–1.47) All-cause mortality: HR model 1: 0.56 (0.43–0.71) HR model 2: 0.79 (0.61–1.03) HR model 3: 0.87(0.66–1.14) Breast-cancer mortality: HR model 2: 0.79 (0.54–1.16) Non-breast cancer mortality: HR model 2: 0.73 (0.5–1.05)	Model 1 (minimally adjusted): age (at diagnosis), total energy intake. Model 2 (medium-adjusted): model 1 + race and ethnicity, education, menopausal status, cancer stage, ER and PR status, physical activity, smoking, HER2 status. Model 3 (maximally adjusted): model 2 + BMI, surgery type, chemotherapy, radiation, HT
Karavasiloglou et al., 2019 [19]	Switzerland and USA	Cohort	110/110	24-h dietary recall and 9-component MD score [26]	Recruit 1988–1994, end of follow-up on 31 December 2011, mean 14.2 years (SEM 0.8)	MDS: 5–9	MDS: 0–4	All-cause mortality: HR model 1: 0.47 (0.29–0.76) HR model 2: 0.78 (0.47–1.32)	Model 1 (minimally adjusted): age (questionnaire completion), race/ethnicity. Model 2 (maximally adjusted): model 1 + race and ethnicity, total energy intake, BMI, moderate-to-vigorous physical activity, smoking, marital status, socioeconomic status, history of menopausal-hormone-therapy used, period since cancer diagnosis, prevalent chronic diseases
Kim et al., 2011 [38]	USA	Cohort	6367/2729	FFQ and aMED-diet score (adapted from 9-components MD score, aMEDs) [26]	Recruitment period 1978–1998, end of follow-up in June 2004	aMEDS Quintile 5	aMEDS Quintile 1	All-cause mortality: RR model 1: 0.74 (0.55–0.99) RR model 2: 0.87 (0.64–1.17) Breast-cancer mortality: RR model 1: 1.11 (0.74–1.66) RR model 2: 1.15 (0.74–1.77) Non-breast cancer mortality: RR model 1:0.58 (0.38–0.88) RR model 2: 0.8 (0.5–1.26)	Model 1 (minimally adjusted): age, time since diagnosis. Model 2 (maximally adjusted): model 1 + race and ethnicity, energy, BMI, physical activity, smoking, menopausal status, cancer stage, physical activity, smoking, treatment (chemotherapy, radiation, TAM), oral-contraceptive use, postmenopausal-hormone-therapy use, multivitamin usage at first birth and parity, alcohol intake, weight change
**QoL**									
**Study**	**Country of** **Study**	**Study** **Design**	**Sample Size/Number in Analysis**	**Dietary** **Assessment and/or MD Adherence** **Assessment**	**Duration/** **Follow-up**	**Exposure**	**Comparator**	**Main result** **(Mean, SD)**	**Variables Used for Adjustment**
Long Parma et al., 2022 [46]; (Zuniga et al., 2019 [47]; Ramirez et al., 2017 [48])	USA	RCT	I: 76, C: 77/ I: 60, C: 65	14-item PREDIMED questionnaire [50]	6 months/ 12 months	PREDIMED 6-month mean score (SD): 8.7 (0.3) Individualised anti-inflammatory dietary prescriptions and behaviour change, 6-month monthly workshops, 12-month monthly navigation, motivational interviewing and tailored newsletters	PREDIMED 6-month mean score (SD): 7.6 (0.3) Minimal nutritional information and two telephone calls prior to assessment appointments.	FACT-G ^a^: *p* = 0.41 6-month: I 87.96 (12.48), C 84.47 (15.81) 12-month: I 85.21 (13.38), C 84.57 (16.42) FACT-G subscales: Social Well-Being *p* = 0.77 6-month: I 20.96 (5.37), C 20.54 (5.94) 12-month: I 20.77 (5.15), C 20.46 (6.17) Emotional Well-Being *p* = 0.76 6-month: I 20.91 (2.75), C 19.97 (3.67) 12-month: I 20.22 (3.23), C 19.76 (3.98) Functional Well-Being *p* = 0.98 6-month: I 21.76 (4.29), C 20.71 (5.24) 12-month: I 20.60 (4.68), C 20.78 (5.42) Physical Well-Being *p* = 0.62 6-month: I 24.13 (3.91), C 23.25 (4.42) 12-month: I 23.60 (4.09), C 23.57 (4.13) BCS ^b^: *p* = 0.82 6-month: I 25.01 (5.38), C 24.15 (5.86) 12-month: I 24.77 (5.34), C 24.31 (6.37) CES-D ^c^: *p* = 0.51 6-month: I 2.45 (2.18), C 2.65 (2.39) 12-month: I 2.85 (2.74), C 2.88 (2.70) Perceived Stress Scale ^d^: *p* = 0.01 Baseline: I 21.77 (7.63), C 19.75 (7.60) 6-month: I 20.64 (7.61), C 20.32 (8.31) 12-month: I 21.59 (7.44), C 20.01 (8.23) (*p* = 0.019 for main effect in I: reduction between baseline and 6-month)	None
Alvarez-Bustos et al., 2021 [39]; (Ruiz-Casado et al., 2020 [40])	Spain	Cross- sectional	180/180	14-item PREDIMED questionnaire [50]	NA	MDS > 7	MDS ≤ 7	Cancer related fatigue: No strong evidence for an association between adherence to the MD and cancer-related fatigue (numerical results were not reported)	None
Barchitta et al., 2020 [41]	Italy	Cross- sectional	68/68	14-item PREDIMED questionnaire [50]	NA	PREDIMED ≥10 positive items	PREDIMED ≤5 positive items	No strong evidence for an association between MD adherence and overall QoL or QoL subscales (EORTC QLQ-C30 ^c^) (numerical results were not reported)	None
Porciello et al., 2020 [42]; (Porciello et al., 2019 [43])	Italy	Cross- sectional	309/309	14-item PREDIMED questionnaire [50]	NA	PREDIMED > 7	PREDIMED ≤ 7	EORTC QLQ-C30 ^e^ subscales: Physical functioning: MDH 83.3 (14.5), MDL 78.9 (17.8), *p* = 0.02 β-model 1: 0.199, *p* = 0.001 β-model 2: 0.207, *p* = 0.001 β-model 3: 0.169, *p* = 0.006 Pain: MDH 23.1 (21.7), MDL 28.5 (24.3), *p* = 0.04 β-model 1: −0.175, *p* = 0.002 β-model 2: −0.174, *p* = 0.005 β-model 3: −0.131, *p* = 0.027 Dyspnoea: β-model 1: −0.115, *p* = 0.045 Insomnia: β -model 1: −0.114, *p* = 0.048 β -model 2: −0.131, *p* = 0.029 EQ-5D-3L Scale ^f^: MDH 0.87 (0.11), MDL 0.84 (0.12), *p* = 0.05 β-model 1: 0.167, *p* = 0.004 β-model 2: 0.190, *p* = 0.003 (Results in other subscales and EORTC QLQ-B23 ^e^ are presented in Supplementary Materials Table S7)	Model 1: age, cancer stag. Model 2: age, cancer stage, BMI, type of surgery, comorbidities, combined therapy. Model 3: age, cancer stage, smoking status, step count, education, civil status (married or single)
**Health-Related Parameters**									
Skouroliakou et al., 2017 [49]	Greece	RCT	I: 35, C: 35/ I: 26, C: 24	FFQ and 0–9 score (revised to include fish intake) [25]	6 months	MDS at 6 months mean (SD): 7.65 (0.68) Personalized dietary intervention based on MD and physical-activity recommendations from ACS	MDS at 6 months mean (SD): 4.44 (1.04) Updated American Cancer Society Guidelines	BMI (kg/m^2^): *p* = 0.97 I 27.55 (4.69), C 27.73 (5.7) Body weight (kg): *p* = 0.89 I 72.69 (13.83), C 72.53 (15.61) Waist circumference (cm): *p* = 0.48 I 94.36 (11.37), C 96.97 (13.06) Blood glucose (mg/dL): *p* < 0.002 (ANCOVA *p* = 0.01) I 91.03 (9.96), C 105.95 (21.04) TC (mg/dL): *p* = 0.62 I 203.83 (44.56), C 209.15 (36.36) LDL-C (mg/dL): *p* = 0.56 I 123.18 (46.73), C 130.78 (34.39) HDL-C (mg/dL): *p* = 0.08 I 66.52 (17.56), C 57.36 (13.83) TAG (mg/dL): *p* = 0.86 I 89 (61.13), C 86.79 (43.74)	Blood-glucose levels adjusted for BMI and estimated weekly MET-mins in ANCOVA analysis
Lorenzo et al., 2020 [45]	Spain	Cross- sectional	90/67	FFQ and 12 questions from the 14-item PREDIMED questionnaire [50]	NA	PREDIMED (12 questions) >7	PREDIMED (12 questions) ≤7	BMI (kg/m^2^): *p* ≥ 0.05 MDH 27.8 (3.2), MDL 28.3 (5.7) Body weight (kg): *p* ≥ 0.05 MDH 68.9 (8.9), MDL 72.3 (14.1) Waist circumference (cm): *p* ≥ 0.05 MDH 87.8 (9.1), MDL 91.7 (15.3) Hip circumference (cm): *p* ≥ 0.05 MDH 106.3 (11.7), MDL 104.7 (11.7) Waist to hip ratio: *p* ≥ 0.05 MDH 0.82 (0.14), MDL 0.87 (0.18) Prevalence of obesity: *p* ≥ 0.05 MDH 68.9%, MDL 80%	Age and BMI
Negrati et al., 2021 [44]	Italy	Cross- sectional	139/80	Diet score (range 0–55) [51]	NA	Diet score (range 0–55). Quartile 4: mean 38	Diet score (range 0–55). Quartile 1: mean 28.5	BMI (kg/m^2^): r = −0.110, *p* ≥ 0.05 MDH 29.3 (6.30), MDL 30.8 (6.20) Blood glucose (mg/dL): r = −0.216, *p* ≥ 0.05 MDH 85.3 (14.72), MDL 91.2 (17.32) Insulin: r = −0.20, *p* ≥ 0.05 MDH 8.7 (11.282), MDL 12.8 (4.69) HOMA-IR: r = −0.176, *p* ≥ 0.05 MDH 1.92 (3.05), MDL 3.06 (1.25) TC (mg/dL): r = −0.024, *p* ≥ 0.05 MDH 239.1 (31.08), MDL 230 (94.94), LDL-C (mg/dL): r = −0.192, *p* ≥ 0.05 MDH 132.4 (34.74), MDL 148.8 (33.09) HDL-C (mg/dL): r = −0.02, *p* ≥ 0.05 MDH 60.3 (10.58), MDL 59.1 (13.97) TAG (mg/dL): r = 0.11, *p* ≥ 0.05 MDH 143 (62.39), MDL 135.5 (68.44)	None
Porciello et al., 2020 [42]; (Porciello et al., 2019 [43])	Italy	Cross- sectional	309/309	14-item PREDIMED questionnaire [50]	NA	PREDIMED > 7	PREDIMED ≤ 7	BMI (kg/m^2^): MDH 27.21 (6.13), MDL 28.24 (5.97)	None

aMEDS: Alternative Mediterranean Diet Score; BC: breast cancer; BMI: body-mass index; C: control group; CNT: cannot tell; ER: estrogen receptor; FFQ: Food Frequency Questionnaire; HDL-C: high-density-lipoprotein cholesterol; HER-2: human epidermal growth factor receptor 2; HOMA-IR: Homeostatic Model Assessment of Insulin Resistance; HR: hazard ratio; HT: hormonal therapy; I: intervention group; LDL-C: low-density-lipoprotein cholesterol; MD: Mediterranean diet; MDH: high-MD-adherence group; MDL: low-MD-adherence group; MDS: Mediterranean Diet Score; NA: not applicable; NR: not reported; PR: progesterone receptor; QoL: quality of life; RCT: randomised controlled trial; RR: relative risk; SD: standard deviation; SEM: standard error of the mean; TAG: triacylglycerol; TAM: tamoxifen; TC: total cholesterol; WC: waist circumference; 95% CI: 95% confidence interval. ^a^: FACT-G (Functional Assessment of Cancer Therapy–General): a 27-item questionnaire (range 0–108) designed to measure four domains of QoL in cancer patients, physical (range 0–28), social (range 0–28), emotional (range 0–24) and functional well-being (range 0–28). The higher the score, the better the QoL [52]. ^b^: BCS: breast cancer subscale (range 0–40) of FACT-B (FACT-G + BCS). The higher the score, the better the QoL [53]. ^c^: CES-D (Centre for Epidemiologic Studies Depression Scale): the higher the score, the greater frequency and number of depression symptoms (range 0–60) [54]. ^d^: Perceived Stress Scale: the higher the score, the higher the stress (range 0–56: 0–18 low stress, 19–37 moderate stress, 38–56 high stress) [55]. ^e^: EORTC QLQ-C30 (European Organization for Research and Treatment of Cancer Quality-of-Life Questionnaire Core 30) and EORTC QLQ-BC23 (breast-cancer module): include functional scales (a high score for a functional scale represents a high/healthy level of functioning), symptom scales and single items (a high score for a symptom scale/item represents a high level of symptomatology/problems) and a global health status/QoL scale (a high score represents a high QoL), range 0–100 for all of the scales individual items) [56]. ^f^: EQ-5D-3L (European Quality of Life 5 Dimensions 3 Level): comprises the following five dimensions: mobility, self-care, usual activities, pain/discomfort and anxiety/depression. The digits for the five dimensions can be combined into a five-digit number and converted to a single summary index, with higher scores indicating higher health utility (0: a health state equivalent to death; negative: worse than death; 1: perfect health) [57].

## Data Availability

The data presented in this study are available in Supplementary Materials.

## Supplementary Materials

The following supporting information can be downloaded at: https://www.mdpi.com/article/10.3390/nu15092099/s1. Table S1: PRISMA checklist; Table S2: Search strategy in detail; Table S3: Adapted Newcastle–Ottawa scale; Table S4: Table of screened full-text results; Table S5: Characteristics of included studies; Table S6: GRADE assessment; Table S7: The QoL results reported by Porciello et al., 2020 [42] (Porciello et al., 2019 [43]); Figure S1: Meta-analysis all-cause mortality (medium adjusted); Figure S2: Meta-analysis BMI (random-effects model).

## Systematic Review Registration

PROSPERO CRD42022318559 https://www.crd.york.ac.uk/PROSPERO/display_record.php?RecordID=318559.
