# Supplementary material for: The Role of the Mediterranean Diet in Breast Cancer Survivorship: A Systematic Review and Meta-Analysis of Observational Studies and Randomised Controlled Trials
Source: Nutrients. 2023 Apr 27;15(9):2099. doi: 10.3390/nu15092099 (PMC10180628; doi:10.3390/nu15092099)

Supplementary number and description	Page
**Supplementary Table S1:** PRISMA checklist	2-3
**Supplementary Table S2:** Search strategy in detail	4
**Supplementary Table S3:** Newcastle–Ottawa scale<br>(cohort study and adapted for cross-sectional studies)	5-6
**Supplementary Table S4:** List of excluded papers (at full-text screening stage)	8-10
**Supplementary TableS5:** Characteristics of included studies	11-14
**Supplementary Table S6:** GRADE assessment	15-16
**Supplementary Table S7:**The QoL result of Porciello et al., 2020 [42]	17-19
**Supplementary Figure S1:** Meta-analysis all-cause mortality (medium adjusted)	20
**Supplementary Figure S2:** Meta-analysis BMI (Random-effects model)	21

Supplementary Table S1. PRISMA checklist

Section/topic	#	Checklist item	Reported on page #
**TITLE**			
Title	1	Identify the report as a systematic review, meta-analysis, or both.	Title page
**ABSTRACT**			
Structured summary	2	Provide a structured summary including, as applicable: background; objectives; data sources; study eligibility criteria, participants, and interventions; study appraisal and synthesis methods; results; limitations; conclusions and implications of key findings; systematic review registration number.	Abstract and p1
**INTRODUCTION**			
Rationale	3	Describe the rationale for the review in the context of what is already known.	p2
Objectives	4	Provide an explicit statement of questions being addressed with reference to participants, interventions, comparisons, outcomes, and study design (PICOS).	p2
**METHODS**			
Protocol and registration	5	Indicate if a review protocol exists, if and where it can be accessed (e.g., Web address), and, if available, provide registration information including registration number.	Abstract and p 2
Eligibility criteria	6	Specify study characteristics (e.g., PICOS, length of follow-up) and report characteristics (e.g., years considered, language, publication status) used as criteria for eligibility, giving rationale.	p3-5
Information sources	7	Describe all information sources (e.g., databases with dates of coverage, contact with study authors to identify additional studies) in the search and date last searched.	p4 and Supplement: Search Strategy
Search	8	Present full electronic search strategy for at least one database, including any limits used, such that it could be repeated.	p4 Supplement: Search Strategy
Study selection	9	State the process for selecting studies (i.e., screening, eligibility, included in systematic review, and, if applicable, included in the meta-analysis).	p4-5
Data collection process	10	Describe method of data extraction from reports (e.g., piloted forms, independently, in duplicate) and any processes for obtaining and confirming data from investigators.	p4-5
Data items	11	List and define all variables for which data were sought (e.g., PICOS, funding sources) and any assumptions and simplifications made.	p3-5
Risk of bias in individual studies	12	Describe methods used for assessing risk of bias of individual studies (including specification of whether this was done at the study or outcome level), and how this information is to be used in any data synthesis.	p5
Summary measures	13	State the principal summary measures (e.g., risk ratio, difference in means).	p5-6
Synthesis of results	14	Describe the methods of handling data and combining results of studies, if done, including measures of consistency (e.g., I^2) for each meta-analysis.	p5-6

Section/topic	#	Checklist item	Reported on page #
Risk of bias across studies	15	Specify any assessment of risk of bias that may affect the cumulative evidence (e.g., publication bias, selective reporting within studies).	p5-6 and Supplement: Adapted Newcastle-Ottawa scale
Additional analyses	16	Describe methods of additional analyses (e.g., sensitivity or subgroup analyses, meta-regression), if done, indicating which were pre-specified.	p5-6
**RESULTS**			
Study selection	17	Give numbers of studies screened, assessed for eligibility, and included in the review, with reasons for exclusions at each stage, ideally with a flow diagram.	p6-7, Figure 1 and Supplement: List of excluded papers (at full-text screening stage)
Study characteristics	18	For each study, present characteristics for which data were extracted (e.g., study size, PICOS, follow-up period) and provide the citations.	p6-7, and Supplement: TableS5 Characteristics of included studies
Risk of bias within studies	19	Present data on risk of bias of each study and, if available, any outcome level assessment (see item 12).	p7-9, Table 2 and Supplement: GRADE assessment
Results of individual studies	20	For all outcomes considered (benefits or harms), present, for each study: (a) simple summary data for each intervention group (b) effect estimates and confidence intervals, ideally with a forest plot.	p8-20, Figure 2, Table 3 and Supplement: Table S7
Synthesis of results	21	Present results of each meta-analysis done, including confidence intervals and measures of consistency.	p8-20, Figure 2, Figure 3, Figure 4 and Supplement: GRADE assessment, Figures S1, Figure S2
Risk of bias across studies	22	Present results of any assessment of risk of bias across studies (see Item 15).	p7-8, Table 2 and Supplement: GRADE assessment
Additional analysis	23	Give results of additional analyses, if done(e.g., sensitivity or subgroup analyses, meta-regression [see Item 16]).	p20, Figure 3, Figure 4 and Supplement: Figures S1, Figure S2
**DISCUSSION**			
Summary of evidence	24	Summarize the main findings including the strength of evidence for each main outcome; consider their relevance to key groups (e.g., healthcare providers, users, and policy makers).	p17-20
Limitations	25	Discuss limitations at study and outcome level (e.g., risk of bias), and at review-level (e.g., incomplete retrieval of identified research, reporting bias).	p19
Conclusions	26	Provide a general interpretation of the results in the context of other evidence, and implications for future research.	p20
**FUNDING**			
Funding	27	Describe sources of funding for the systematic review and other support (e.g., supply of data); role of funders for the systematic review.	p20

Supplementary Table S2. Search strategy

Medline search terms:

1. exp Breast Neoplasms/
2. ((breast* or mammary) adj3 (cancer* or carcinoma* or neoplasm* or tumor* or tumour*)).mp. [mp=title, abstract, original title, name of substance word, subject heading word, floating sub-heading word, keyword heading word, organism supplementary concept word, protocol supplementary concept word, rare disease supplementary concept word, unique identifier, synonyms]
3. 1 or 2
4. Diet, Mediterranean/
5. (mediterranean adj2 (diet* or lifestyle)).mp. [mp=title, abstract, original title, name of substance word, subject heading word, floating sub-heading word, keyword heading word, organism supplementary concept word, protocol supplementary concept word, rare disease supplementary concept word, unique identifier, synonyms]
6. 4 or 5
7. 3 and 6
8. limit 7 to humans

Embase search terms:

1. exp breast cancer/ or breast tumor/
2. ((breast* or mammary) adj3 (cancer* or carcinoma* or neoplasm* or tumor* or tumour*)).mp. [mp=title, abstract, heading word, drug trade name, original title, device manufacturer, drug manufacturer, device trade name, keyword heading word, floating subheading word, candidate term word]
3. 1 or 2
4. exp Mediterranean diet/
5. (Mediterranean adj2 (diet* or lifestyle)).mp. [mp=title, abstract, heading word, drug trade name, original title, device manufacturer, drug manufacturer, device trade name, keyword heading word, floating subheading word, candidate term word]
6. 4 or 5
7. 3 and 6
8. limit 7 to human

Web of science search terms:

1. TS=(Mediterranean Near/s(diet* or lifestyle))
2. TS=((breast* or mammary) NEAR/3(cancer* or carcinoma* or neoplasm* or tumor* of tumour*))
3. 1 and 2

Cochrane Library search terms:

1. MeSH descriptor: [Breast Neoplasms] explode all trees
2. MeSH descriptor: [Diet, Mediterranean] explode all trees
3. ((breast* or mammary) NEAR/3 (cancer* or carcinoma* or neoplasm* or tumor* or tumour*)):ti,ab,kw
4. (mediterranean NEAR/2 (diet* or lifestyle)):ti,ab,kw
5. 1 OR 3
6. 2 OR 4
7. 5 AND 6

Supplementary Table S3: **Newcastle–Ottawa scale**
(cohort study and adapted for cross-sectional studies)

NEWCASTLE - OTTAWA QUALITY ASSESSMENT SCALE
COHORT STUDIES

Note: A study can be awarded a maximum of one star for each numbered item within the Selection and Outcome categories. A maximum of two stars can be given for Comparability

Selection (maximum 4 points):

1) Representativeness of the exposed cohort
   a) truly representative of the average _______________ (describe) in the community ✸
   b) somewhat representative of the average ______________ in the community ✸
   c) selected group of users eg nurses, volunteers
   d) no description of the derivation of the cohort

2) Selection of the non exposed cohort
   a) drawn from the same community as the exposed cohort ✸
   b) drawn from a different source
   c) no description of the derivation of the non exposed cohort

3) Ascertainment of exposure
   a) secure record (eg surgical records) ✸
   b) structured interview ✸
   c) written self report
   d) no description

4) Demonstration that outcome of interest was not present at start of study
   a) yes ✸
   b) no

Comparability (maximum 2 points):

1) Comparability of cohorts on the basis of the design or analysis
   a) study controls for _____________ (age and ER/cancer subtype/cancer stage) ✸
   b) study controls for any additional factor ✸ (This criteria could be modified to indicate specific control for a second important factor.)

Outcome (maximum 3 points)

1) Assessment of outcome
   a) independent blind assessment ✸
   b) record linkage ✸
   c) self report
   d) no description

2) Was follow-up long enough for outcomes to occur

a) yes (select an adequate follow up period for outcome of interest) ✵
b) no

3) Adequacy of follow up of cohorts

a) complete follow up - all subjects accounted for ✵

b) subjects lost to follow up unlikely to introduce bias - small number lost - > 20_ % (select an adequate %) follow up, or description provided of those lost) ✵

c) follow up rate < 80_% (select an adequate %) and no description of those lost

d) no statement

Studies were considered to have
Low risk of bias: 3 - 4 points in selection domain AND 1 -2 points in comparability domain AND 2 - 3 stars in outcome/exposure domain;
Medium risk of bias: 2 points in selection domain AND 1 - 2 points in comparability domain AND 2 - 3 stars in outcome/exposure domain;
High risk of bias: 0 - 1 point in selection domain OR 0 point in comparability domain OR 0 - 1 point in outcome domain.

NEWCASTLE - OTTAWA QUALITY ASSESSMENT SCALE
Adapted for CROSS-SECTIONAL STUDIES

Note: A study can be awarded a maximum of one star for each numbered item within the Selection and Outcome categories. A maximum of two stars can be given for Comparability

Selection (maximum 5 points)

1) Representativeness of the exposed cohort

a) Truly representative of the average in the target population. (all subjects or random sampling) ✵
b) Somewhat representative of the average in the target group. * (non-random sampling) ✵
c) Selected group of participants/convenience sample
d) No description of the sampling strategy or the derivation of included participants

2) Sample size

a) Justified and satisfactory (including sample size calculation).✵
b) Not justified
c) No information provided

3) Non-respondents:

a) Comparability between respondents and non-respondents characteristics is established, and the response rate is satisfactory. ✵

b) The response rate is unsatisfactory, or the comparability between respondents and non-respondents is unsatisfactory.

c) No information provided on the response rate or the characteristics of the responders and the non-responders

4) Ascertainment of the exposure (risk factor):

a) Validated measurement tool. ✵✵

b) Non-validated measurement tool, but the tool is available or described. ✵

c) No description of the measurement tool

Comparability (maximum 2 points):

1) The subjects in different outcome groups are comparable, based on the study design or analysis. Confounding factors are controlled.

a) The study controls for the most important factor (age and ER/cancer subtype/cancer stage). ✵

b) The study control for any additional factor. ✵

Outcome (maximum 3 points)

1) Assessment of outcome

a) independent blind assessment ✵✵

b) record linkage or Unblinded assessment using objective validated laboratory methods or medical diagnosis✵✵

c) self report ✵

d) no description

2) Statistical test:

a) The statistical test used to analyse the data is clearly described and appropriate, and the measurement of the association is presented, including confidence intervals and the probability level (p value). ✵

b) The statistical test is not appropriate, not described or incomplete.

Studies were considered to have

Low risk of bias: 4 - 5 points in selection domain AND 1 -2 points in comparability domain AND 2 - 3 points in outcome/exposure domain;

Medium risk of bias: 2 - 3 points in selection domain AND 1 - 2 points in comparability domain AND 2 - 3 points in outcome/exposure domain;

High risk of bias: 0 - 1 point in selection domain OR 0 point in comparability domain OR 0 - 1 point in outcome domain.

Supplementary Table S4. List of excluded papers (at full-text screening stage)

No	Author (Year)	G CHEN	Reason for exclusion	JZ NIU	Reason for exclusion	Final decision	Final reason for exclusion
**1**	Augustin et al. (2017)	exclude	Background article, ineligible intervention: MD+x VS MD	exclude	MD is not a variable in this study	exclude	Wrong intervention (MD in both groups)
**2**	Bernstein et al. (2019)	maybe	abstract	exclude	abstract	exclude	No full text report
**3**	Biasini et al. (2015)	maybe	Abstract, full paper found	exclude	Abstract	exclude	No full text report (Full text found doesn't fully match this abstract)
**4**	Biasini et al. (2016)	maybe	Abstract, full paper found	exclude	Abstract	exclude	No full text report (Full text found doesn't fully match this abstract)
**5**	Bruno et al. (2015)	exclude	No MD evaluation	exclude	No association of MD was reported	exclude	Wrong intervention
**6**	Bruno et al. (2021a)	exclude	No MD evaluation	exclude	MD is not an independent intervention	exclude	Wrong intervention
**7**	Bruno et al. (2018)	exclude	ineligible intervention: MD+PA VS non	exclude	Wrong population	exclude	Wrong intervention
**8**	Bruno et al. (2021b)	exclude	involved both ovarian cancer and BC patients	exclude	Wrong population	exclude	Wrong population
**9**	Bruno et al. (2020)	exclude	BC patients were involved in the population, not stratified	exclude	Wrong population	exclude	Wrong population

Table S4 continued

No	Author (Year)	G CHEN	Reason for exclusion	JZ NIU	Reason for exclusion	Final decision	Final reason for exclusion
**10**	Calabrese et al. (2019)	exclude	ineligible intervention: MD+x VS MD, full paper found	exclude	abstract	exclude	Wrong intervention
**11**	Cioffi et al. (2020)	maybe	Ask for full text	exclude	abstract	exclude	No full text report
**12**	Cortesi et al. (2021)	exclude	MD was presumed, no MD evaluation	exclude	MD is not an intervention in this study	exclude	Wrong intervention
**13**	Farina et al. (2021)	maybe	Check full text	exclude	Population is not clear	exclude	No contact information of authors were found<br>No full text report
**14**	Flynn and Reinert (2010)	exclude	no MD evaluation	exclude	MD is not an intervention in this study	exclude	Wrong intervention
**15**	George et al. (2014)	exclude	BC patients were involved in the population, not stratified	exclude	Wrong population	exclude	Wrong population
**16**	Golubic et al. (2018)	exclude	No control group<br>BC patients were involved in the population, not stratified	exclude	Wrong population	exclude	Wrong population
**17**	Huang et al. (2018)	exclude	No MD evaluation	exclude	No MD mentioned	exclude	No MD evaluation
**18**	Koh et al. (2019)	exclude	No MD evaluation	exclude	Exposure not related to MD	exclude	No MD evaluation
**19**	Kwon et al. (2020)	exclude	No control group	maybe	Only the data in MD group was reported, outcome is miRNA	exclude	No control group<br>Wrong study design
**20**	Lagiou et al. (2006)	exclude	BC patients is not clear	exclude	Wrong population	exclude	Wrong population
**21**	Laudisio et al. (2021)	maybe	abstract	exclude	Detailed information needed	exclude	No full text report

Table S4 continued

No	Author (Year)	G CHEN	Reason for exclusion	JZ NIU	Reason for exclusion	Final decision	Final reason for exclusion
**22**	Lopez-Pentecost et al. (2022)	exclude	Wrong population, not BC patient	exclude	Wrong population	exclude	Wrong population
**23**	Montagnese et al. (2020)	exclude	No control	exclude	No control	exclude	No control group<br>Wrong study design
**24**	Nct (2019)	maybe	Trial not yet completed?	exclude	Study not complete	exclude	Study not completed<br>(No reply from author)<br>No full text report
**25**	Park et al. (2022)	exclude	Not BC patient	exclude	Wrong population	exclude	Wrong population
**26**	Pierce et al. (2007)	exclude	No MD evaluation	exclude	MD is not the intervention	exclude	Wrong intervention
**27**	Pistelli et al. (2021)	exclude	No MD evaluation<br>No control group	exclude	MD is not an intervention	exclude	Wrong study design
**28**	Roldan-Jimenez et al. (2022)	exclude	No control group<br>Wrong outcome (MD adherence)	exclude	MD is not an independent intervention	exclude	No control group<br>Wrong study design
**29**	Stefani et al. (2019)	exclude	No PA in control	exclude	MD is not an independent intervention	exclude	Wrong intervention
**30**	Trestini et al. (2021)	exclude	No control group	exclude	Before after	exclude	No control group<br>Wrong study design
**31**	Ubaidullah et al. (2021)	exclude	Wrong population (no stratified BC data), No MD evaluation	exclude	Wrong population	exclude	Wrong population
**32**	Villarini et al. (2012)	exclude	No MD evaluation	exclude	MD is not an independent intervention	exclude	Wrong intervention
**33**	Whalen et al. (2017)	exclude	Wrong population, not BC patients	exclude	Population is not BC patients	exclude	Wrong population

Supplementary Table S5. Characteristics of included studies

Study	Country of study	Age	Ethnicity	Education	Smoking	BMI (kg/m2)	Postmenopausal (n (%))	Breast Cancer Stages	Breast cancer subtypes	Time since breast cancer diagnosis at recruitment	Time since last treatment	Previous Treatment
Alvarez-Bustos et al., 2021; Ruiz-Casado et al. (2020)	Spain	Mean (SD): 51(9)	NR	Primary or less 13%, secondary 35%, College 52%	Smoker 11%	26 (4.4)	NR (by AI :25%)	Stage I 35% Stage II 49% Stage III 16%	NR	Days 856 (1950)	Treatment during study data collection: Trastuzumab and HT was allowed	Chemotherapy 73% Anthracyclines 68% Trastuzumab 20% Radiotherapy 65% Hormonotherapy 81% (Aromatase inhibitors 25%)
Barchitta et al., 2020	Italy	36-68	Italian	NR	NR	NR	NR	Stage I-III	NR	NR	At least 6 months prior to the recruitment	Radiotherapy or chemotherapy treatment
Di Maso et al., 2020	Italy	Median: 55 (range: 23-78)	Italian	<7 years 50.5%,7-11years 28.6%,>12years 20.5%	Current smoker: 19.96%	<25 : 55.2%, 25-29.9: 31.9%, ≥30: 11.8%	900 (61.94%)	Stage I 32.69% Stage II 44.7% Stage III-IV 13.28%	ER and/or PR+ 20%, ER and PR- 41.5%	No longer than 1 year	NR	NR
Ergas et al., 2021	USA	Mean (SD): 59.7 (11.9) Range: 24-94	White: 68.1% Black: 6.6% Asian/Pacific Islander: 13.0% Hispanic: 10.3% American Indian/Alaska Native: 2.1%	High school or less 547 (14.9%), Some college 1245 (34.0%), College graduate 1024 (28.0%), Postgraduate 842 (23.0%), Unknown 2 (0.1%)	Never 2092 (57.2%), Former 1408 (38.5%), Current 154 (4.2%), Unknown 6 (0.2%)	Q1-Q5 (by ACS quintile range): 29.9(7.3)-26.3(5.2)	2600 (71.0%)	Stage I 54.9% Stage II 34.2% Stage III 9.5% Stage IV 1.5%	ER+ 83.9% ER- 16.0% Unknown 0.1%	Mean: 2.3 months (range: 0.7-18.7)	NR	Surgery 96.7%, Chemotherapy 46.7%, Radiotherapy 44.4% Hormonotherapy 74.7%

Table S5 continued

Study	Country of study	Age	Ethnicity	Education	Smoking	BMI (kg/m2)	Postmenopausal (n (%))	Breast Cancer Stages	Breast cancer subtypes	Time since breast cancer diagnosis at recruitment	Time since last treatment	Previous Treatment
Karavasiloglou et al., 2019	Switzerland / USA	Mean: 62.4 (SEM 1.6)	Non-Hispanic white 91.6 % Non-Hispanic black 5.0% Mexican-American 1.5% Other 1.9%	NR	Never: 42.5% , Former: 40.5% , Current: 16.9%	Mean (SEM): 26.4(0.5)	NR	NR	NR	Mean: 8.6 years (SEM 0.7)	NR	NR
Kim et al., 2011	USA	30-55	NR	NR	Current smoker : Q1 vs Q5 22.3%, 7.7%	aMED Q1 vs Q5: mean 26 vs 25.1	CNT	aMED Q1 vs Q5 Stage I: 57.1% vs 56.8% Stage II: 35% vs 33.9% Stage III: 7.9% vs 9.3%	NR (by TAM: aMEDs Q1 vs Q5 64.1% vs 60.2%)	At least 12 months after breast cancer diagnosis (diet measurement)	NR	aMED Q1 vs Q5: Chemotherapy 36% vs 36.1%, Radiotherapy 39.5% vs 45% Tamoxifen 64.1% vs 60.2%
Long Parma et al., 2022; Zuniga et al. (2019); Ramirez et al. (2017)	USA	Total Mean: 56.6<br><br>Mean (SD) I: 55.28 (9.85), C: 57.86 (8.81)	Anglo 42.4%, Latino 51.2% Other 6.4%	High school graduate or less 16 (12.8%) Some college/Assoc degree 41 (32.8%) College graduate or higher 68 (54.4%)	NR	Overweight or Obese, Mean (SD) I: 31.2(4.1) C: 32.7(5.2)	NR	Stage 0: 9.6%, Stage I: 28%, Stage II: 30.4%, Stage III: 16.8%, Don't know: 15.2%	NR, (by HT: ER+ 33.6%)	2 or more months	< 6 months: 12.8%, 6-24 months: 24%, ≥24 months 64%	Surgery 93.6%, Chemotherapy 65.6%, Radiotherapy 61.6%, HT 33.6%

Table S5 continued

Study	Country of study	Age	Ethnicity	Education	Smoking	BMI (kg/m2)	Postmeno pausal (n (%))	Breast Cancer Stages	Breast cancer subtypes	Time since breast cancer diagnosis at recruitment	Time since last treatment	Previous Treatment
Lorenzo et al., 2020	Spain	Mean (SD): High MD 57.9 (7.3) Low MD: 53.7 (11.4)	Spanish	NR	NR	<25:28.9%, ≥ 25:71.1%	68.90%	Stage I/II 64.4% Stage III/IV 14.4% Unknown 21.1%	ER+ 76.7%, ER - 13.3%, unknown 10%	newly diagnosed	NR	NR
Negrati et al., 2021	Italy	Mean (SD) 54.9 (10.6)	NR	NR	NR	MDS Quartile1 vs Quartile 4: mean 30.8 vs 29.3	NR	Stage 0 8.8% Stage I 45.2% Stage II 40.2% Stage III 6.3%	NR	NR	at least 2 months	NR
Porciello et al., 2020; Porciello et al. (2019)	Italy	Mean (SD): 52 (9.2)	Italian	≤11years 111 (36%),≥12 years 197(64%)	Never 152 (49.1%), Former 95 (30.7%), Current 58 (18.8 %)	Mean (SD) 27.6 (6.0), <25: 41.1%, 25.0–29.9: 28.5%, ≥30.0: 30.4%	NR	Stage I 30.1% Stage II 55.6% Stage III 14.3%	NR (by HT: ER+ 53.7%)	Within 12 months	Treatment during study data collection: Chemotherapy 16.1%, Radiotherapy 7.4% Hormonothera py 52.4%, Biological therapy 14.9%	Surgery 99.4%, Chemotherapy 46%, Radiotherapy 46.3% Hormonothera py 1.3%, Biological therapy 0.3%
Skouroliakou et al., 2017	Greece	Mean (SD) I: 51.49 (8), C:52.17 (11.52)	NR	NR	Current: Intervention 9 (31.4%); Control 5 (14.2%)	Mean (SD) I: 27.55(4.69) C: 27.73(5.7)	I:16 (45.7%), C: 18(51.4%)	Stage I-IIIA	NR, (by HT: ER+ 72%)	up to 3 month	NR	Chemotherapy 76%, Radiotherapy 66%, HT 68%

ACS: American Cancer Society; AI: Aromatase inhibitors; aMEDS: alternative Mediterranean Diet Score; BC: Breast cancer; BMI: Body mass index; C: control group; CNT: Can not tell; ER: estrogen receptor; FFQ: Food Frequency Questionnaire; HER-2: human epidermal growth factor receptor 2; HT: Hormonal therapy; I: intervention group; MD: Mediterranean diet; MDS: Mediterranean Diet Score; NA: Not applicable; NR: Not reported; PR: progesterone receptor; Q: quintile; QoL: quality of life; RCT: Randomised controlled trial; SD: standard deviation; SEM: standard error of the mean; TAM: Tamoxifen;

Supplementary Table S6. GRADE assessment

Certainty assessment							№ of patients		Effect		Certainty
№ of studies	Study design	Risk of bias	Inconsistency	Indirectness	Imprecision	Other considerations	High MD adherence	Low MD adherence	Relative (95% CI)	Absolute (95% CI)	
**All-cause mortality**											
3	observational studies	not serious	not serious	not serious	not serious	none	272/1447 (18.8%)*	302/1175 (25.7%) *	**HR 0.78** (0.66 to 0.93)	**50 fewer per 1,000** (from 79 fewer to 16 fewer)	⨁⨁◯◯ Low
**Breast cancer mortality**											
2	observational studies	not serious	not serious	not serious	serious[a]	none	177/1423 (12.4%)	168/1104 (15.2%)	**HR 0.82** (0.65 to 1.03)	**26 fewer per 1,000** (from 50 fewer to 4 more)	⨁◯◯◯ Very low
**Non-breast cancer mortality**											

Certainty assessment							№ of patients		Effect		Certainty
№ of studies	Study design	Risk of bias	Inconsistency	Indirectness	Imprecision	Other considerations	High MD adherence	Low MD adherence	Relative (95% CI)	Absolute (95% CI)	
2	observational studies	not serious	not serious	not serious	serious[b]	none	97/1423 (6.8%)	137/1104 (12.4%)	**HR 0.67** (0.50 to 0.90)	**39 fewer per 1,000** (from 60 fewer to 12 fewer)	⨁◯◯◯ Very low
**BMI**											
3	observational studies	serious[c]	not serious	not serious	not serious	none	208	208	-	MD **0.93 kg/m2 lower** (2.03 lower to 0.17 higher)	⨁◯◯◯ Very low

CI: confidence interval; HR: hazard Ratio; MD: mean difference

Explanations:

a. 95%CI includes a HR of 1.0 and HR under 0.75, which represents wide CI

b. Total number of event does not meet optimal information size criteria

c. Low quality by NOS

* One study (Karavasiloglou et al., 2019) did not report case number

Supplementary Table S7. QoL findings in the study of Porciello et al., 2020

Study	Country of study	Study design	Sample size/ Number in analysis	Dietary assessment & MD adherence assessment	Exposure & Comparator	Main result (Mean ,SD)	
Porciello et al., 2020; (Porciello et al., 2019)	Italy	Cross-sectional	309/309	14-item PREDIMED questionnaire (Martínez-González et al., 2012)	PREDIMED>7 & PREDIMED≤7	EQ-5D-3L Score [a]:<br>MDH 0.87 (0.11), MDL 0.84 (0.12), p=0.05<br>β -model1**: 0.167, p=0.004<br>β -model2****: 0.190, p=0.003<br>β -model3******: 0.169, p=0.063<br>EQRTC QlQ-C30 [b] subscales:<br>Physical functioning:<br>MDH 83.3 (14.5), MDL 78.9 (17.8),p=0.02<br>β -model1*: 0.199, p=0.001<br>β -model2**: 0.207, p=0.001<br>β -model3***: 0.169, p=0.006<br>Role functioning:<br>MDH 80 (22.8), MDL 78.5 (24.3), p=0.56<br>β -model1*: 0.060, p=0.296<br>β -model2**: 0.052, p=0.382<br>β -model3***: 0.037, p=0.534<br>Emotional functioning:<br>MDH 75.3 (251.6), MDL 71.8 (21.2), p=0.15<br>β -model1*: 0.067, p=0.247<br>β -model2**: 0.059, p=0.973<br>β -model3***: 0.033, p=0.587<br>Cognitive functioning:<br>MDH 80.8 (21.5), MDL 81.4 (21.7), p=0.82<br>β -model1*: 0.067, p=0.247<br>β -model2**: 0.059, p=0.973<br>β -model3***: 0.033, p=0.587	Constipation:<br>MDH 15 (21.4), MDL 14.3 (23.5), p=0.81<br>β -model1*: -0.013, p=0.827<br>β -model2**: -0.09, p=0.787<br>β -model3***: -0.037, p=0.552<br>Diarrhoea:<br>MDH 7.4 (15.7), MDL 10 (17.4), p=0.16<br>β -model1*: -0.033, p=0.568<br>β -model2**: -0.039, p=0.515<br>β -model3***: -0.021, p=0.717<br>Financial impact:<br>MDH 19 (27.3), MDL 19.1 (27.1), p=0.97<br>β -model1*: -0.036, p=0.540<br>β -model2**: 0.005, p=0.937<br>β -model3***: -0.021, p=0.717<br>Global Health Status/Qol:<br>MDH 62.9 (22.1), MDL 63.2 (20.6), p=0.93<br>β -model1*: 0.010, p=0.856<br>β -model2**: 0.024, p=0.695<br>β -model3***: 0.032, p=0.603<br>Social functioning:<br>MDH 76.9 (25.9), MDL 75.7 (25.7), p=0.67<br>β -model1*: 0.028, p=0.630<br>β -model2**: 0.020, p=0.741<br>β -model3***: 0.0004, p=0.950

Table S7 (continued)

Study	Country of study	Study design	Sample size/ Number in analysis	Dietary assessment & MD adherence assessment	Exposure & Comparator	Main result (Mean ,SD)
						Nausea and vomiting:<br>MDH 7.8 (13.3), MDL 6.9 (14.2), p=0.6<br>β -model1*: 0.019, p=0.742<br>β -model2**: 0.015, p=0.802<br>β -model3***: 0.049, p=0.407<br>Pain:<br>MDH 23.1 (21.7), MDL 28.5 (24.3), p=0.04<br>β -model1*: -0.175, p=0.002<br>β -model2**: -0.174, p=0.005<br>β -model3***: -0.131, p=0.027<br>Dyspnea:<br>MDH 18.12 (22.9), MDL 21.6 (23.1), p=0.19<br>β -model1*: -0.115, p=0.045<br>β -model2**: -0.101, p=0.098<br>β -model3***: -0.069, p=0.249<br>Insomina:<br>MDH 26.7 (28.3), MDL 32.8 (27.6), p=0.06<br>β -model1*: -0.114, p=0.048<br>β -model2**: -0.131, p=0.029<br>β -model3***: -0.096, p=0.101<br>Appetite loss:<br>MDH 6.4 (17.4), MDL 7.6 (15.1), p=0.52<br>β -model1*: -0.033, p=0.564<br>β -model2**: -0.034, p=0.574<br>β -model3***: -0.131, p=0.027<br>Fatigue:<br>MDH 32.9 (23.6), MDL 35 (23.5), p=0.42<br>β -model1*: -0.080, p=0.163<br>β -model2**: -0.075, p=0.217<br>β -model3***: -0.062, p=0.300<br>EQRTC QlQ-B23 [b] subscales<br>Body image:<br>MDH 65.6 (30.6), MDL 60.6 (29.9), p=0.15<br>β -model1*: 0.076, p=0.190<br>β -model2**: 0.065, p=0.294<br>β -model3***: 0.059, p=0.329<br>Sexual functioning:<br>MDH 80.8 (22.1), MDL 81.5 (22.4), p=0.78<br>β -model1*: -0.037, p=0.526<br>β -model2**: -0.034, p=0.584<br>β -model3***: -0.003, p=0.595<br>Future perspective:<br>MDH 45.6 (33.6), MDL 42 (34.1), p=0.36<br>β -model1*: -0.058, p=0.325<br>β -model2**: 0.131, p=0.404<br>β -model3***: 0.131, p=0.524<br>Systematic therapy side effects:<br>MDH 23.9 (18.1), MDL 26.2 (18.9), p=0.29<br>β -model1*: -0.080, p=0.164<br>β -model2**: -0.063, p=0.293<br>β -model3***: -0.038, p=0.531<br>Breast symptoms:<br>MDH 20.2 (18.1), MDL 24.1 (20), p=0.08

β -model1*: -0.095, p=0.086
β -model2**: -0.062, p=0.311
β -model3***: -0.054, p=0.362
Arm symptoms:
MDH 21.1 (19), MDL 21.7 (20.4), p=0.79
β -model1*: -0.073, p=0.210
β -model2**: -0.063, p=0.303
β -model3***: -0.040, p=0.500

a. EQ-5D-3L (European Quality of Life 5 Dimensions 3 Level): comprises the following five dimensions: mobility, self-care, usual activities, pain/discomfort and anxiety/depression, the digits for the five dimensions can be combined into a 5-digit number and converted to a single summary index, with higher scores indicating higher health utility (0: a health state equivalent to death, negative: worse than death, to 1: perfect health

b. EORTC QLQ-C30 (European Organization for Research and Treatment of Cancer Quality of Life Questionnaire Core 30) and EORTC QLQ-BC23 (Breast cancer module): include functional scales (a high score for a functional scale represents a high/ healthy level of functioning), symptom scales and single items (a high score for a symptom scale/ item represents a high level of symptomatology/ problems) and a global health status/ QoL scale (a high score represents a high QoL), range 0-100 for all of the scales/single-item);
*Model 1: age, cancer stage;
**Model 2: age, cancer stage, BMI, type of surgery, comorbidities, combined therapy;
***Model 3: age, cancer stage, smoking status, step count, education, civil status (married or single)

Supplementary Figure S1. Meta-analysis of MD adherence and all-cause mortality (medium adjusted)

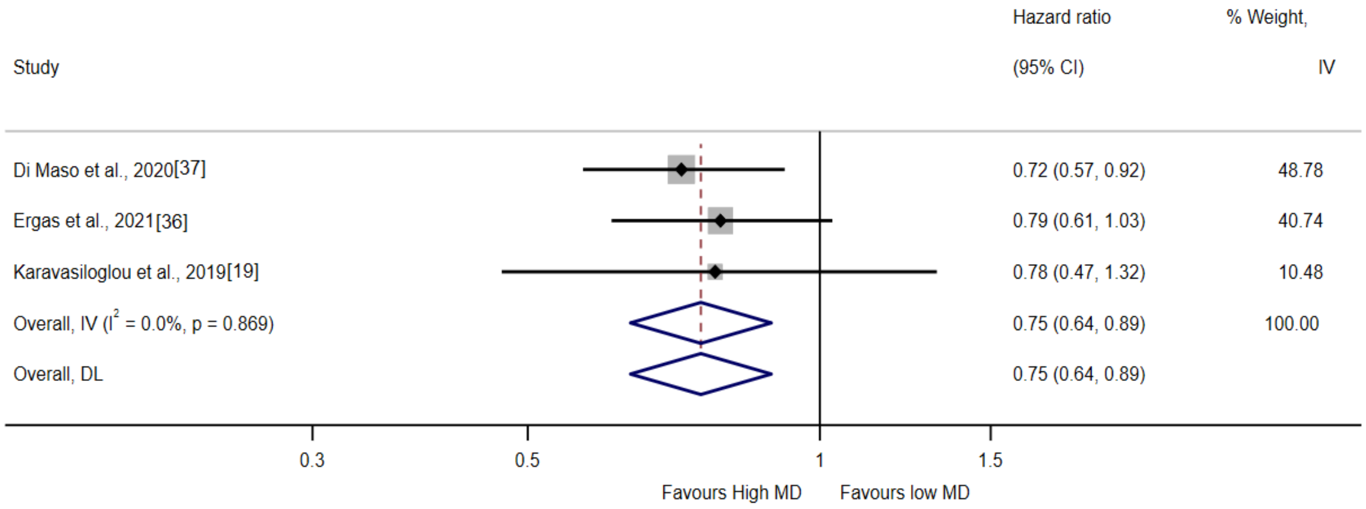

IV: Weights are from fixed-effects model; DL: Weights are from random-effects model
MD: Mediterranean diet

Supplementary Figure S2. Meta-analysis of MD adherence and BMI (random-effects model)

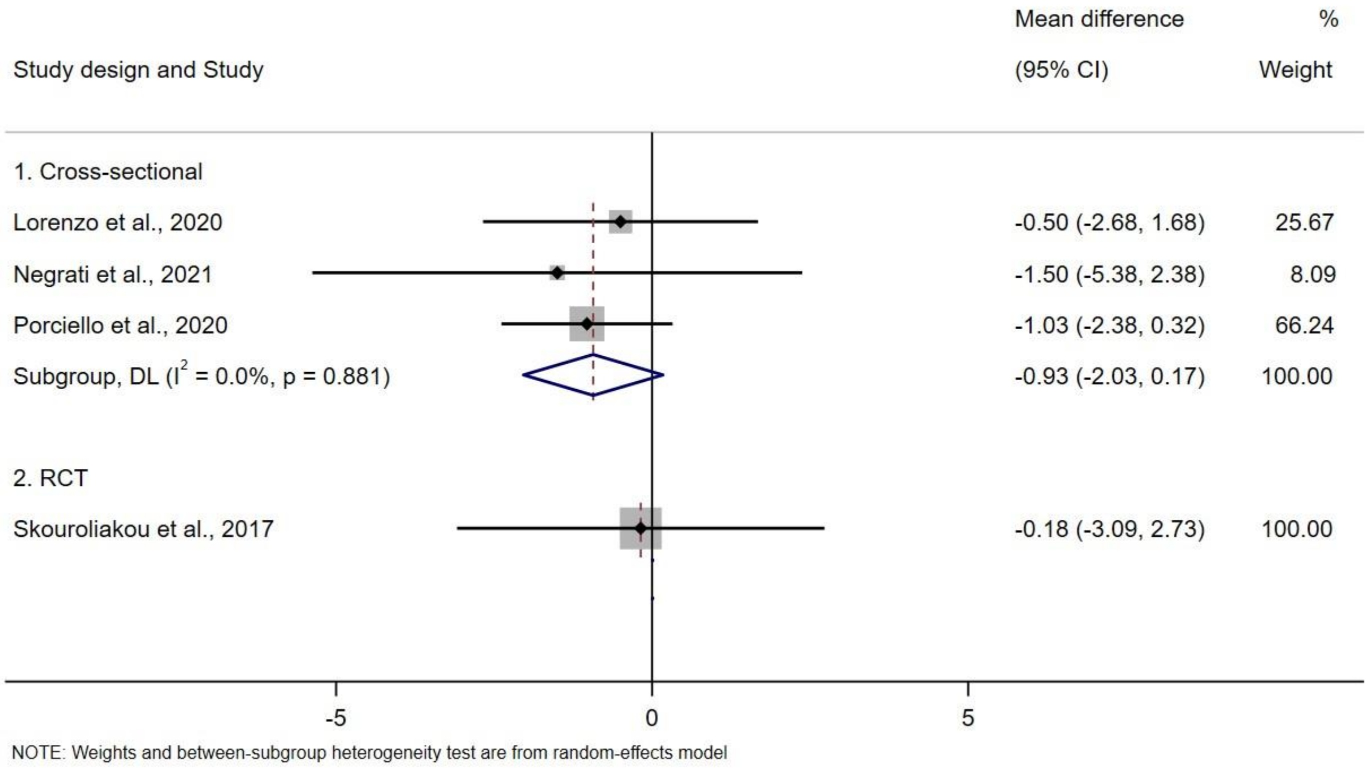

Supplement: Supplementary file 1 [file nutrients-15-02099-s001.zip › nutrients-2306110-supplementary.pdf]
